# V-NOTES hysterectomy under spinal anaesthesia: A pilot study

**DOI:** 10.52054/FVVO.14.3.040

**Published:** 2022-09-30

**Authors:** E.C. Gündoğdu, E Mat, Y Aboalhasan, G Yıldız, G Başol, K Tolga Saraçoğlu, G Arslan, A Kale

**Affiliations:** Department of Obstetrics and Gynecology, University of Health Science KartalDr. Lutfi Kirdar City Hospital, Istanbul, Turkey; Division of Gynecologic Oncology, Department of Obstetrics and Gynecology, University of Health Science Kartal Dr Lutfi Kirdar City Hospital, Istanbul, Turkey; Department of Obstetrics and Gynecology, Siirt Training and Research Hospital, Siirt, Turkey; Department of Anesthesiology and Reanimation, University of Health Science Kartal Dr Lutfi Kirdar City Hospital, Istanbul, Turkey

**Keywords:** Hysterectomy, Laparoscopy, Spinal anaesthesia, V-NOTES

## Abstract

**Background:**

Spinal anaesthesia has not been widely adopted for laparoscopic surgeries until now. There are a few studies that have shown that spinal anaesthesia is at least as safe as general anaesthesia. The need for additional analgesics can be reduced by utilising early postoperative analgesic effects of spinal anaesthesia, and maximum benefit can be obtained from minimally invasive approaches when V-NOTES surgery is performed under spinal anaesthesia.

**Objective:**

Combining V-NOTES with spinal anaesthesia to improve minimally invasive surgical techniques and provide maximum benefit to patients.

**Materials and Methods:**

Patients who were found to have benign pelvic organ pathologies, required a hysterectomy and were considered suitable for V-NOTES hysterectomy under spinal anaesthesia were included in this study. Spinal anaesthesia was achieved with 12.5 mg 0.5% hyperbaric bupivacaine in the sitting position. Perioperative events and complications related to spinal anaesthesia were noted. Postoperatively, the pain was evaluated using a visual analogue scale at the 6th, 12th, and 24th hours.

**Main outcome measures:**

To evaluate the feasibility and safety of spinal anaesthesia in VNOTES hysterectomy and to increase the advantages of minimally invasive surgical procedures. Results: No conversion to conventional laparoscopy or laparotomy was required in all six operated patients. Conversion from spinal anaesthesia to general anaesthesia was unnecessary, and no major perioperative incident occurred in any of the cases.

**Conclusion:**

In the current study by our team, we demonstrated that V-NOTES hysterectomy could be performed safely under spinal anaesthesia in well-selected patients. The need for additional analgesics can be reduced by utilising early postoperative analgesic effects of spinal anaesthesia, and maximum benefit can be obtained from minimally invasive approaches when VNOTES surgery is performed under spinal anaesthesia.

**What is new?:**

V-NOTES hysterectomy could be performed safely under spinal anaesthesia in well-selected patients.

## Introduction

Hysterectomy is one of the most frequently performed major gynaecological operations worldwide. Abdominal hysterectomy, laparoscopic hysterectomy, and vaginal hysterectomy may be the preferred surgical approach when surgery is indicated (Whiteman et al., 2008). Recent studies have demonstrated the increasing popularity of laparoscopic hysterectomy over the past 20 years (Wright et al., 2013), and transvaginal natural orifice transluminal endoscopic surgery (V-NOTES) has been introduced as a combination of conventional vaginal and laparoscopic surgery (Kaouk et al., 2009). It has been an approach adopted by gynaecologists with the developing technological innovations. It has become the shining star of laparoscopic gynaecological surgeries over the years. Its advantages include less postoperative pain, no abdominal wall infection, and no scar or incisional hernia (Su et al., 2012; Lee et al., 2014; Baekelandt, 2015; Kale et al., 2017).

While developments in minimally invasive surgeries have continued in recent years, several studies have confirmed many advantages of spinal anaesthesia, including less postoperative pain, a lower incidence of nausea and vomiting, and an earlier ability to ambulate (Liu et al., 2005; Capdevila and Dadure, 2004). However, laparoscopic hysterectomy is routinely performed under general anaesthesia regardless of the transabdominal or transvaginal route. This is generally explained by the possibility of impaired respiratory function due to the pneumoperitoneum, Trendelenburg position during laparoscopic gynaecological surgery, or the patient’s inability to tolerate the surgery.

Although not widely accepted, there are reports of laparoscopic hysterectomy performed successfully under regional anaesthesia, while there are no reports of the use of spinal anaesthesia in V-NOTES hysterectomy (Sinha et al., 2008; Moawad et al., 2018). We have aimed to evaluate the feasibility and safety of spinal anaesthesia in V-NOTES hysterectomy and to increase the advantages of minimally invasive surgical procedures.

## Materials and methods

The study was conducted in accordance with the Declaration of Helsinki guidelines in a tertiary referral hospital in Istanbul, Turkey, between January 2019 and June 2020. The local hospital’s ethics committee gave ethics approval (Reference Number: 2020/514/178/20, Approved 27 May 2020). The study design was explained to all patients before they were included. A detailed medical history was obtained from all patients. Abdominal examination, bimanual examination and transvaginal ultrasonography, and, if necessary, abdominal ultrasonography were performed on all patients. The inclusion criteria were as follows: patients aged 30 to 70 years, with American Society of Anesthesiologists (ASA) physical status I-II, with pelvic organ pathologies associated with the uterus, cervix and/or ovaries that would require a hysterectomy, and eligible to perform V-NOTES hysterectomy. Patients with contraindications for pneumoperitoneum or spinal anaesthesia, a history of tubo-ovarian abscess, a history of deep endometriosis, suspected severe pelvic adhesions, a nodule in the Pouch of Douglas, a fixed uterus, and sexually inactive patients were excluded from the study. Patients with uterine prolapse who could undergo vaginal hysterectomy were also excluded from the study. Two anaesthesiologists evaluated all patients regarding their suitability for spinal anaesthesia, and detailed information was given to the patients about the anaesthesia procedure. The same teams performed both surgery and spinal anaesthesia. In our clinic, the recommendations of the Enhanced Recovery After Surgery Society guideline are applied to ensure optimal perioperative care (Altman et al., 2020). Preoperative bowel preparation was not used. ECG, arterial blood pressure, and pulse oximetry were evaluated. After obtaining vital signs,10 mL/ kg of Ringer’s lactate solution was given to all patients for 30 minutes. Premedication was not used in any of the patients. Patients were placed in a sitting position. The subarachnoid space was entered through the L4-L5 space with a sharp- tipped 25G spinal needle. Spinal anaesthesia was achieved with 12.5 mg 0.5% hyperbaric bupivacaine in the sitting position. The patient was then placed in the supine position. Motor block was evaluated using the Bromage scale. Sensory block level was evaluated with a pinprick test. All patients received an intravenous dose of 0.05 mg/ kg of midazolam. During the first 20 minutes, the patients’ blood pressure was followed at 5 minute intervals and measured at 10 minute intervals. As in other types of surgery, oxygen supplementation was given to all patients by oral and nasal mask. Pain monitoring was followed by Visual Analogue Scale (VAS) scores. When VAS was > 3, it was planned to administer 1-1.5 mg/kg IV tramadol with 1 g paracetamol and repeat it every 12 hours if necessary.

After the anaesthesia procedure, the patients were placed in the dorsal lithotomy position. To reduce the incidence of postoperative infection, 2 g of cefazolin was administered intravenously 15 minutes before the incision. Povidone iodine solution was used as a topical antiseptic in the surgical field. The surgical field was covered with a sterile drape. 18-French Foley catheter was placed in the urethra. A transvaginal port system (Alexis; Applied Medical Resources Corp., Rancho Santa Margarita) was used to perform a V-NOTES hysterectomy. The Alexis wound retractor was placed in the vagina, and four self-retaining sleeves were placed in the GelSeal cap. Then the GelSeal cap was placed over the Alexis wound retractor. CO2 was insufflated from this port to a pressure of 15 mmHg at a flow rate of 0.4 L/min. After the pneumovagina was created, the vagina and cervix were visualised with a 10 mm 30-degree endoscope. Before the colpotomy incision, the operating table was tilted to the Trendelenburg position 10 degrees to reduce the risk of bowel injury. A circumferential incision was made around the cervix using an ultrasonic scalpel system (Harmonic HD 1000i shears, 5-mm diameter; Ethicon). A bladder flap was created by cutting the visceral peritoneum to isolate the bladder from the lower uterine segment. The posterior peritoneal fold was found and dissected using an ultrasonic scalpel system. This allowed access to the Douglas pouch. Anterior and posterior incisions were extended up transversely across the cervix. The pneumoperitoneum was created at a pressure of 8 to 12 mmHg CO2, and the abdominal cavity was visualised. After entering the abdominal cavity, the Trendelenburg position was changed to 20 degrees as needed. Uterosacral ligament complexes, uterine vessels, leaflets of the broad ligaments, utero-ovarian ligaments and round ligaments were identified and dissected using an electrothermal bipolar vessel sealing device (LigaSure, 5 mm diameter, blunt tip; Covidien). The uterus was freed from all attachments. If salpingectomy is to be performed, the Fallopian tube is identified and cut using an electrothermal bipolar vessel closure device (LigaSure, 5 mm diameter, blunt type; Covidien). Fallopian tubes were taken out of the abdomen and left in the outer part of the wound retractor. If an adnexectomy is performed, the infundibulopelvic ligament is identified and cut using an electrothermal bipolar vessel closure device (LigaSure, 5 mm diameter, blunt tip; Covidien) similar to other ligaments. Adnexae were taken out of the abdomen and left in the outer part of the wound retractor. After adequate haemostasis was achieved, the uterus was removed from the vagina. The vaginal cuff was closed vaginally using a single coated Vicryl suture (90 cm, polyglactin 910; Ethicon EndoSurgery).

The patients’ age, primary complaint, menopausal status, systemic diseases, indication for hysterectomy, and pre and postoperative biochemical and haematological parameters were recorded. The operation time was recorded from the beginning of the colpotomy incision to the vaginal closure. Perioperative events and complications related to spinal anaesthesia like nausea, vomiting, headache, and shoulder pain, were noted. Postoperatively, the pain was evaluated using a visual analogue scale at the 6th, 12th, and 24th hours. When the patients came to the gynaecological inpatient service, they were given liquid food, and solid food was recommended 2 hours after the operation. Patients were discharged 24 hours after the surgery.

## Results

**Figure 1:**
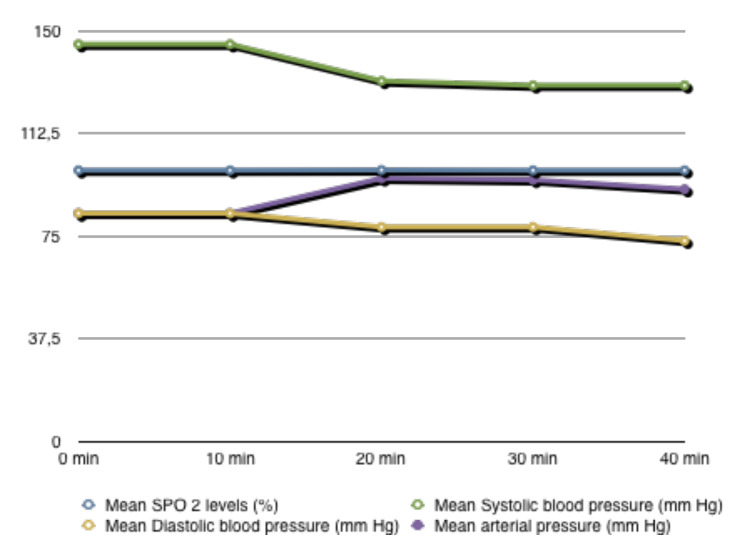
Chart showing changes in patients’ mean arterial pressure, mean SPO 2 levels, mean systolic blood pressure, mean diastolic blood pressure during surgery.

V-NOTES hysterectomy was performed under spinal anaesthesia in six patients who met the previously mentioned criteria and gave written informed consent. The mean patient age was 49 years (min = 43, max = 55, standard deviation [SD] = 4.04 years), and the mean BMI was 25.9 kg/ m2 (min = 24.7, max = 27.3, SD = 0.93 kg/m2). All patients were multiparous (median = 3.1; min = 2, max = 5). Two patients had hypertension, and one had diabetes mellitus (Table I). One patient had a history of cholecystectomy and umbilical hernia repair, and another had a history of breast cancer. The indication for hysterectomy was abnormal uterine bleeding in all patients. There was no significant change in the patients’ mean arterial pressure, mean SPO2 levels, mean systolic blood pressure, and mean diastolic blood pressure during the surgery (Figure 1). The patients were informed about the possible advantages and disadvantages of salpingectomy and salpingo-oophorectomy. Bilateral salpingo-oophorectomy was performed in 4 patients, and bilateral salpingectomy was performed in 3 patients. The median uterine weight was 135 g (range, 90 - 200). 3 patients had uterine fibroids. The largest fibroid size was 3 cm. No conversion to conventional laparoscopy or laparotomy was required. Conversion from spinal anaesthesia to general anaesthesia was not needed, and no major perioperative incident occurred in any of the cases. Nausea was observed in one patient, and shoulder pain was observed in another towards the end of the operation, and both resolved spontaneously without needing medical treatment.

**Table I:** Characteristics of the patients, indication for hysterectomy, histopathological results and weight of the uterus sample.

Case no.	Age (years)	Parity	BMI (kg/m2)	Comorbidity	Indication for hysterectomy	Operation	Hystopathological result	Uterus weight (grams)
1	55	5	26,5	Nil	Abnormal uterine bleeding	V-NOTES hysterectomy and bilateral salpingo oophorectomy	No significant pathology seen	90
2	43	2	24,7	Nil	Abnormal uterine bleeding	V-NOTES hysterectomy and bilateral salpingectomy	Leiomyoma and adenomyosis	200
3	50	3	25,4	DM	Abnormal uterine bleeding	V-NOTES hysterectomy and bilateral salpingo oophorectomy	Leiomyoma	150
4	51	3	27,3	Nil	Abnormal uterine bleeding	V-NOTES hysterectomy and bilateral salpindo oophorectomy	No significant pathology seen	110
5	48	3	26,2	HT	Abnormal uterine bleeding	V-NOTES hysterectomy and bilateral salpingo oophorectomy	No significant pathology seen	130
6	47	3	25,4	HT	Abnormal uterine bleeding	V-NOTES hysterectomy and bilateral salpingectomy	Leiomyoma	140

BMI: Body mass index, DM: Diabetes Mellitus, HT: Hypertension, V-NOTES: Transvaginal Natural Orifice Transluminal Endoscopic Surgery.

The average operating time was 58 min (SD = 10.5 min).

**Figure 2:**
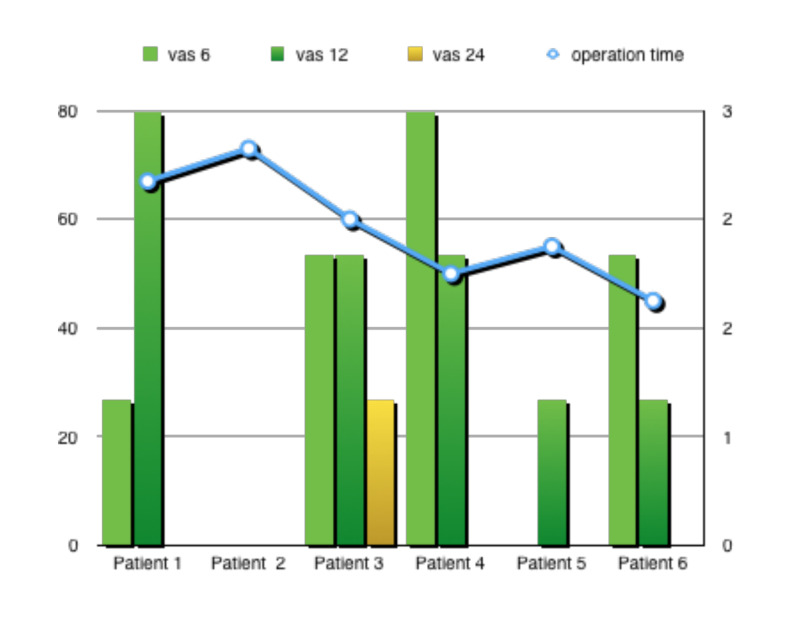
Showing changes in patients’ visual analoge scale scores and operation time.

All patients were mobilised at the 4th postoperative hour. The mean postoperative VAS pain scores at the 6th, 12th, and 24th hours were 1.3 (range, 0-3), 1.5 (range, 0-3), and 0.1 (range, 01), respectively (Figure 2). Two patients experienced postoperative nausea that responded to Granisetron administration. Vomiting, severe shoulder pain, or headache was not recorded. Blood loss was minimal, and none of the patients required blood transfusion. The mean haemoglobin level change was 0.18 g/ dL (SD = 0.14 g/dL) on postoperative day 1. All patients were discharged at the postoperative 24th hour. All patients were followed up one week and one month after the operation. None of the patients had complaints such as headaches or back pain related to spinal anaesthesia. No patient was diagnosed with postoperative cuff cellulitis, cuff separation, or bleeding.

## Discussion

To date, this is the first study to evaluate the feasibility and safety of V-NOTES hysterectomy under spinal anaesthesia. Interestingly, despite the increasing interest in minimally invasive approaches, regional anaesthesia still plays a minor role, and general anaesthesia has become the dominant or even the only approach in laparoscopic gynaecological surgeries. Although upper abdominal laparoscopic surgery under regional anaesthesia has been reported in many studies, only a few have been published on laparoscopic pelvic surgery under spinal anaesthesia (Donmez et al., 2017; Tzovaras et al., 2008). The main reason spinal anaesthesia is not preferred is the compelling effects of laparoscopic surgeries on respiratory and cardiac function.

Studies evaluating respiratory function in various laparoscopic abdominal surgeries performed under spinal anaesthesia give different success rates according to the operation type (Donmez et al., 2017; Tzovaras et al., 2008). Many factors, such as performing the surgery in the upper or lower abdomen, intra-abdominal pressure during the operation, and the operation time, may affect respiratory and cardiac function during surgery. It has been shown that measurable changes in haemodynamic parameters due to insufflation and patient position during laparoscopy are not reflected in clinic parameters when a pressure of 15 mmHg is not exceeded (Grabowski and Talamini, 2009). In cases where the pneumoperitoneum is created with a pressure of 15 mmHg, there is a 27% decrease in respiratory system compliance, and prolonged duration of pneumoperitoneum may result in a long time to reverse changes in pulmonary compliance (Rauh et al., 2001). Trendelenburg position can be used in most laparoscopic pelvic surgeries to facilitate visualisation of the pelvic region and perform a successful operation. If the Trendelenburg position is preferred, the respiratory function may adversely be affected, and it may be difficult to complete the surgery under spinal anaesthesia. However, adverse effects on respiratory function may be milder when V-NOTES is performed, as intra-abdominal pressure is maintained at lower levels compared to conventional laparoscopy. Based on these points, we think keeping the intra- abdominal pressure between 8 to 12 mmHg and the short operation time in patients who underwent V-NOTES hysterectomy under spinal anaesthesia are the most important factors preventing adverse respiratory effects. It may also be important to keep the Trendelenburg position below 20 degrees during the operation.

In addition to respiratory effects, numerous studies have shown that laparoscopic pelvic surgery also has some cardiac effects. These cardiac effects are thought to be related to the decrease in venous return to the inferior vena cava due to the increased intra-abdominal pressure during laparoscopic surgery, resulting in decreased cardiac output and, ultimately, hypotension (Grabowski and Talamini, 2009). Another factor affecting the changes caused by laparoscopy in the cardiovascular system is the patient’s position. Studies show that the decrease in cardiac output in patients with pneumoperitoneum is more pronounced in the head-up position and tends to increase in the Trendelenburg position (Williams and Murr, 1993; Junghans et al., 1997). In our study, no significant change in mean arterial pressure, hypotension, or respiratory function impairment was observed in any patients. In our study, we consider that the intra-abdominal pressure not exceeding 12 mmHg during the operation and the short duration of the operation are important reasons for the absence of significant adverse cardiac effects. In addition, as we mentioned before, the Trendelenburg position may have played a positive role in cardiac function with its tendency to increase cardiac output.

One of the reasons for the increasing popularity of minimally invasive approaches is the concerns and difficulties experienced in the management of acute postoperative pain after abdominal surgery (Andreae and Andreae, 2012). It has been shown that adequate analgesia cannot be achieved in half of the patients using routine pain control methods (Gandhi et al., 2011). Moreover, the lack of pain control after surgery may delay the patient’s recovery. It has been shown in many studies that pain scores are lower, and the need for analgesics is less in the early postoperative period after spinal anaesthesia compared to general anaesthesia. (Wang et al., 1996; Massicotte et al., 2009; Kessous et al., 2012). In patients who have undergone surgery under spinal anaesthesia, it can be expected that lower abdominal pain will be felt less in the first few hours with the ongoing effect of spinal anaesthesia after the operation. In V-NOTES surgery, there is no abdominal incision that may cause pain. However, patients describe some pain in the pelvic region in the early hours after surgery, and spinal anaesthesia can reduce the sensation of pain caused by a vaginal incision in the first hours after the operation. In our study, none of the patients who underwent V-NOTES hysterectomy under spinal anaesthesia had a VAS score above 3, and none needed analgesics.

One of the conditions that can be encountered in surgeries performed under spinal anaesthesia is shoulder pain, which may limit the use of spinal anaesthesia in laparoscopic surgeries. Shoulder pain occurs as a result of the stretching of the diaphragm with CO2 insufflation and is transmitted through the cervical roots, which are not affected by spinal anaesthesia. Once shoulder pain occurs, it may resolve on its own or be treated with medication, or it may be severe enough to require a conversion from spinal anaesthesia to general anaesthesia. The incidence of shoulder pain specific to laparoscopic gynaecological surgery is unknown due to studies’ paucity. However, it is known that the probability of shoulder pain increases as the duration of pneumoperitoneum increases or the Trendelenburg degree increases. To reduce the possibility of shoulder pain, we aimed to keep the pneumoperitoneum time and Trendelenburg degree to a minimum without compromising surgical safety. In our study, only one patient had mild shoulder pain that did not require medication towards the end of the operation.

There are only a few studies examining laparoscopic gynaecological surgery under spinal anaesthesia. One of these few studies is a case of total laparoscopic hysterectomy that Moawad et al. (2018) successfully performed under regional anaesthesia (Moawad et al., 2018). In another study, Singh et al. (2015) evaluated and reported the use of combined spinal and epidural anaesthesia for conventional laparoscopic surgeries with 50 patients. In that study, eight out of 50 patients underwent laparoscopy-assisted vaginal hysterectomy or total laparoscopic hysterectomy under combined anaesthesia. Conversion to general anaesthesia was required in only two patients due to severe shoulder pain, and no complications were reported (Singh et al., 2015). Conversion from spinal anaesthesia to general anaesthesia was not required in our study. When it comes to V-NOTES hysterectomy, there is a need to consider its position against vaginal hysterectomy. Vaginal hysterectomy is considered the first line approach when a hysterectomy is needed for a benign indication (National Institute for Health and Care Excellence, 2018; American Association of Gynecologic Laparoscopists, 2011). However, the choice of hysterectomy route may be influenced by many factors, such as the size and shape of the vagina and uterus, accessibility of the uterus, the need for concurrent procedures, and surgeon training and experience (American College of Obstetricians and Gynecologists’ Committee on Practice Bulletins, 2017). Vaginal hysterectomy may not always be possible in cases of the undescended and immobile uterus or narrow vaginal apex. (Kovac, 2004). An endoscopic approach may be preferred in cases where vaginal hysterectomy cannot be performed. V-NOTES is a very comfortable and safe minimally invasive surgical method performed through natural orifices with laparoscopic surgical equipment. Most studies to date have focused on the comparison of V-NOTES hysterectomy with other laparoscopic hysterectomies (Michener et al., 2021; Kaya et al., 2021). In a study comparing V-NOTES hysterectomy and vaginal hysterectomy, there was no difference in surgical outcomes between the 2 groups, except for the rate of salpingectomy or adnexectomy (V-NOTES group 100%, vaginal group 60%) (Merlier et al., 2022). Therefore, V-NOTES hysterectomy may offer an advantage over vaginal hysterectomy when adnexal removal is required, but there is a need for further research for comparison.

The vaginal route is considered the most cost- effective surgical approach to hysterectomy since no disposable instruments are used, and hospital stay is relatively short. In a study comparing laparoscopy-assisted vaginal hysterectomy and V-NOTES hysterectomy, the V-NOTES hysterectomy group was found to be more costly due to the wound retractor and bipolar vessel closure device. Although our study did not focus on cost analysis, in our experience, V-NOTES hysterectomy does not appear to be more costly than any laparoscopic surgery. In addition, the use of spinal anaesthesia may reduce the cost further by reducing the use of analgesics and shortening hospital stay (Turkistani et al., 2019). Studies will be needed to assess the cost of V-NOTES hysterectomy under spinal anaesthesia in comparison to general anaesthesia and vaginal hysterectomy.

Patient satisfaction may be one of the subjective indicators in the evaluation of V-NOTES hysterectomy under spinal anaesthesia. When the patients were asked about their opinions about the surgery in the postoperative follow-up, all patients stated that they were very satisfied with the surgery. We believe that establishing a relationship of trust between the patient and the team and informing the patients in detail play a key role. We attribute patient satisfaction to the maintenance of communication with the patient during the operation and the close follow-up of the patient during and after the operation.

## Conclusion

Spinal anaesthesia has not been widely adopted for laparoscopic surgeries until now. It has many advantages, such as reduction in postoperative pain, faster ambulation, and faster recovery. Few studies have shown that spinal anaesthesia is at least as safe and feasible as general anaesthesia. In the current pilot study by our team, we demonstrated that V-NOTES hysterectomy could be performed safely under spinal anaesthesia in well-selected patients. The need for additional analgesics can be reduced due to early postoperative analgesic effects of spinal anaesthesia and maximising the benefit of a minimally invasive approach. There is, however, a need for further research to study the feasibility, safety and cost of V-NOTES in comparison to vaginal hysterectomy, as well as comparison of V-NOTES under spinal or general anaesthesia. We are currently conducting a prospective randomised controlled trial to compare the outcomes of performing a V-NOTES hysterectomy under spinal and general anaesthesia.
